# HER2 overexpression and correlation with other significant clinicopathologic parameters in Ivorian breast cancer women

**DOI:** 10.1186/s12907-018-0081-4

**Published:** 2019-01-17

**Authors:** Nguiessan Alphonse Aman, Brahima Doukoure, Kouadio Donatien Koffi, Baumaney Sylvanus Koui, Zie Cheick Traore, Mohamed Kouyate, Ahoua Benjamin Effi

**Affiliations:** 1 Department of Anatomic Pathology, School of Medicine, Alassane Ouattara University, BP V 18 Bouake, Bouake, Ivory Coast; 2 Department of Anatomic Pathology, School of Medicine, Felix H Boigny University, 01 BP V 34 Abidjan 01, Abidjan, Ivory Coast

**Keywords:** Breast cancer, Estrogen receptor, Human epidermal growth factor receptor 2, Progesterone receptor

## Abstract

**Background:**

The overexpression of HER2 is associated with worse prognosis of breast cancer which responds favourably to anti-HER2 therapy. The objective of this study was to determine the frequency of HER2 and its association with clinicopathologic factors in breast cancer in Ivory Coast.

**Methods:**

The study included 608 patients who were histologically diagnosed with invasive primary breast carcinoma. The immunohistochemistry testing for ER, PR, and HER2 was performed on the formalin fixed paraffin-embedded blocks of breast tissue of these patients. The analysis of variance and the Chi-Square Test were used to examine the association of the HER2 status with clinicopathologic prognostic features.

**Results:**

The average age of patients was 47 ± 11 years. Among 608 patients, 355 (58.4%) were premenopausal. Invasive ductal carcinoma of no specific type (511 cases, 84.1%) was the most frequent histologic type. Grade II tumors were 59.8%. The positivity of ER, PR, and ER/PR was 334 cases (54.9%), 252 cases (41.4%), and 356 cases (58.5%), respectively. HER2 was overexpressed in 105 cases (17.3%). The overexpression of HER2 was significantly correlated with Nottingham grade (*p* = 0.007). No association was observed between HER2 expression and age (*p* = 0.568), menopausal status (*p* = 0.929), histologic type (*p* = 0.666), ER (*p* = 0.137), PR (*p* = 0.396), and ER/PR (*p* = 0.134).

**Conclusion:**

Breast cancer occurs in young women. HER2 status is closely related to Nottingham grade. The immunohistochemical analysis of HER2 has prognostic and therapeutic implications, and thus, contributing to efficient clinical management of patients.

## Background

Breast cancer is the most common malignant tumour and the leading cause of death among women in the world [1, 2]. Breast cancer is the first cancer in women, followed by cervical cancer according to the Cancer Registry of Abidjan in Côte d’Ivoire [1]. HER2 is a proto-oncogene found on chromosome 17q encoding tyrosine kinase receptor located on the surface membrane of the epithelial cells of the breast [3–5]. HER2 acts on epidermal growth factor to control various cellular functions, including cell proliferation, cell survival, and apoptosis [6, 7]. The understanding of the underlying mechanisms of HER2 overexpression in the occurrence of breast cancer has led to the discovery of new anti-HER2 targeted therapies for better management of this disease [5, 8]. HER2 positive patients favourably respond to anti-HER2 targeted therapies [9–11]. The amplification or overexpression of HER2 has prognostic and therapeutic significance [12, 13]. The immunohistochemical analysis of HER2 with ER and PR is a routine clinical practice [14–16] which has recently been integrated into the management of breast cancer patients in Ivory Coast, where the data on HER2 are unknown. The objective of this study was to assess the HER2 status and its association with clinicopathologic factors in women breast carcinomas for better treatment strategy and prognostic prediction.

## Methods

### Patients

This study included a prospective series of 608 patients presenting primary invasive breast carcinomas between November 2013 and December 2016 at the Central Laboratory in Abidjan, Ivory Coast. The study material consisted of core needle biopsies (*n* = 550) and surgical samples (*n* = 58) fixed in 10% formalin. The classical histology techniques using hematoxylin and eosin staining were carried on formalin fixed paraffin-embedded breast tissue blocks. The malignant tumors were classified according to the WHO Classification of breast tumors [17] and graded with the criteria of Elston and Ellis [18]. The selected study features were age, menopausal status, histological type, grade, and status of ER, PR, and HER2. The status of ER, PR, and HER2 was determined by immunohistochemistry upon the formalin fixed paraffin-embedded blocks of breast carcinoma patients.

### Immunohistochemistry and scoring

The immunohistochemical assay was done on deparaffinized formalin-fixed tissue sections (thickness 3 μm) of the samples. The hormonal receptors and HER2 were determined using the Ventana BenchMark (Ventana Medical Systems Inc., Tucson, AZ, USA) and the prediluted antibodies (anti-ER clone SP1 for ER, anti-PR clone 1E2 for PR, and anti-HER2 clone 4B5 for HER2).

Slides were reviewed using light microscopy, and IHC staining results for ER and PR were semiquantitatively assessed based on the staining intensity (weak, moderate, intense) and the percentage of malignant cells immunostained. Slides were graded positive for ER and PR when more than 1% of nuclei of malignant cells were stained [19]. The evaluation criteria of HER2 status were based on the intensity of cell membrane immunostaining and the percentage of membrane positive cells, giving a score range of 0–3+ [15]. HER2 negative (score 0 or 1+) was decided when no staining was observed or membrane staining in less than 10% of tumor cells or a faint partial membrane staining in more than 10% of tumor cells, HER2 equivocal (score 2+) when a weak to moderate complete membrane staining in more than 10% of tumor cells and HER2 positive (score 3+) when a strong complete membrane staining in more than 10% of tumor cells. HER2 equivocal was not evaluated in our study due to the lack of fluorescence in situ hybridization. The IHC scoring of ER, PR, and HER2 was reviewed independently by two pathologists. For the purpose of this study, HER2 equivocal and IHC intensity for ER and PR were not considered. The expression of Ki-67, cytokeratin 5/6 (CK 5/6) and human epidermal growth factor receptor 1 (HER1) were not examined. The triple negative consists of the basal-like type (ER-/PR−/ HER2−/ CK 5/6+ / HER1+) and normal-like type (all five markers are negative) [20]. Herein, the breast cancer subtypes were defined according to the IHC expression of ER/PR and HER2 as follows [21–23]:

- Luminal A (ER/PR+, HER2-): ER+/PR+/HER2-; ER+/PR-/HER2-; ER-/PR+/HER2-;
- Luminal B (ER/PR+, HER2+): ER+/PR+/HER2+; ER+/PR-/HER2+; ER-/PR+/HER2+;
- Non luminal HER2+ (ER-/PR-/HER2+);
- Triple negative (ER-/PR-/HER2-).

#### Statistical analysis

Data were collected in an Excel database from Windows 8 (Microsoft Corporation, Redmond, WA, USA) and analysed in SPSS 20.0 (IBM Corp, Armonk, NY, USA). The analysis of variance (ANOVA) evaluated the relationship between the HER2 status and the patients’age. The Chi-Square Test analyzed the correlation between the HER2 status and the menopausal status, the histological type, and the tumor grade. A probability *p*-value < 0.05 was considered statistically significant. The data reported as means ± standard deviation for ANOVA and frequencies for Chi-Square Test.

## Results

### Characteristics of patients

In this study, 608 women were diagnosed with invasive primary breast carcinoma with a mean age of 47,89 ± 11 years (extremes: 24–90 years). Breast cancer was the most common in premenopausal women (58.4%). Invasive ductal carcinoma of no specific type (IDC-NST) was 84.1%. Grade II carcinomas were 59.8%, followed by 20.7% of grade I and 19.5% of grade III. Among 608 patients, 54.9, 41.5, and 58.5% expressed ER, PR, and ER/PR, respectively. The proportion of patients overexpressing HER2 was 17.3%. The Table 1 shows the frequency of HER2 and clinicopathologic variables of the study population.

**Table 1:** Clinicopathologic characteristics of patientes

Variables	Number of patients N (%)
Age (years)	
Mean ± SD	47.9 ± 11
Extremes	24–90
Menopausal status	
Premenopause	355 (58.4)
Postmenopause	253 (41.6)
Histologic type	
IDC-NST	511(84.1)
Lobular	30 (4.9)
Other	67 (11)
Nottingham grade	
I	100 (19.5)
II	307 (59.8)
III	106 (20.7)
Indetermined	95 (00)
ER	
Positive	334 (54.9)
Negative	274 (45.1)
PR	
Positive	252 (41.5)
Negative	356 (58.5)
HER2	
Positive	105 (17.3)
Negative	472 (77.6)
Equivocal	31(5.1)
ER/PR	
ER/PR+	356 (58.5)
ER/PR-	252 (41.5)
Molecular subtype	
Luminal A	268 (46.4)
Luminal B	68 (11.8)
HER2 enriched	37 (6.4)
Triple negative	204 (35.4)

*ER* Estrogen receptor, *HER2* Human epidermal growth factor receptor 2, *IDC-NST* Invasive ductal carcinoma of no special type, *PR* Progesterone receptor, *SD* Standard deviation

### Relationship between HER2 status and clinicopathologic factors

HER2 status was not significantly associated with age of patients (*p* = 0.568), menopausal status (*p* = 0.929), histologic type (*p* = 0.666), ER (*p* = 0.137), and PR (*p* = 0.396). However, the HER2 positivity was closely related to grade (*p* = 0.007). The combined results of patients with HER2+ grade II and III (86.2%) is slightly greater than that of patients with HER2- grade II and III (78.8%).The Table 2 summarizes the association of HER2 expression with clinicopathologic features in 608 patients.

**Table 2:** Association of HER2 status with clinicopathologic factors in breast cancer patients

Variables	HER2 + *N* = 105(%)	HER2- *N* = 472(%)	p
Mean age ± SD	48.6 ± 9.7	47.9 ± 11	0.568
Menopausal			0.929
< 50 years	56(53.3)	276(58.5)	
≥ 50 years	49(46.7)	196(41.5)	
Histologic type			
IDC-NST	91(86.7)	393(83.3)	0.666
Lobular	5(4.8)	25(5.3)	
Other	9(8.5)	54(11.4)	
Tumor grade			0.007*
I	13(13.8)	83(21.1)	
II	70(74.5)	224(57.0)	
III	11(11.7)	86(21.8)	
ER status			0.137
Positive	64(61.0)	250(53.0)	
Negative	41(39.0)	222(47.0)	
PR status			0.396
Positive	47(44.8)	190(40.3)	
Negative	58(55.2)	282(59.7)	
ER/PR status			0.134
ER/PR+	68(64.8)	268(56.8)	
ER/PR-	37(35.2)	204(43.2)	

*ER* Estrogen receptor, *HER2* Human epidermal growth factor receptor 2, *IDC-NST* Invasive ductal carcinoma of no special type, *PR* Progesterone receptor, *SD* Standard deviation, *p p* value; (*): Statistically significant difference with *p* < 0.05

## Discussion

The IHC evaluation of HER2, ER, and PR is already a great advance and relevant for the adequate clinical management of patients with breast cancer in low-resource countries, particularly in Ivory Coast. The overexpression of HER2 is highly correlated with high risk of recurrence, short overall survival, and high mortality in breast cancer patients. Nevertheless, HER2 positive patients receive anti-HER2 targeted therapy which has considerably improved their prognosis. Our current study helps determine the frequency of HER2 in order to optimize the treatment of this cancer. In our study, the proportion of HER2 was 17.3% and is in agreement with literature data which have reported a variation of 15–30% in early breast cancer [4, 5, 24]. Although our result is similar to the findings reported in Senegal/Nigeria (17%) [25], in Mali (18%) [26], and in Tunisia (18.1%) [27], it is different from the rate found in Uganda (22%) [28], in Angola (23,6%) [29], in Ghana (25.5%) [30] and in the USA (25–33%) [5]. Even if our result was included in the interval rate of HER2, it remains low and could be due to the fixation deficiency, the type of antibody used, and the method of detection of HER2 positivity [13, 15, 31–33]. Interestingly, Mitchell et al. [31] have demonstrated that the variability of HER2 overexpression resulted from the loss of HER2 antigen during the under or prolonged fixation of breast specimen and the different used antibodies. They have shown that the DNA of HER2, analyzed by FISH, was not affected by fixation whatever the storage duration of paraffin-embedded blocks of breast tissue, and therefore, indicating that FISH is more accurate in determining HER2 overexpression than IHC [31]. In addition, Varga et al. [32] have revealed that the rate of HER2+ detected by FISH remained stable, while it significantly changed by IHC in a comparative study between FISH and IHC within 7714 patients over a 12-year period. These relevant remarks highlighted the need not only to control the preanalytical factors (fixation) of the samples and the storage conditions of the blocks but only to conduct a research evaluating the impact of IHC and FISH on HER2 status in our setting. Our patients with HER2+ breast cancer would receive trastzumab associated with chemotherapy (anthracycline-taxane), which reduce the risk of recurrence, and thus, improving their survival as described in other studies [34, 35]. The prognostic and predictive value of HER2 with other prognostic factors, including age, menopausal status, histologic type, grade, ER, and PR are relevant for effective management of patients. The mean age and the menopausal status of our study population were independent from HER2 status, although premenopausal women overexpressed more HER2 than postmenopausal women. Similar results were observed by several authors [36–38]. This lack of correlation is probably due to the relative short life expectancy within women in Subsaharan countries, including Ivory Coast. Moreover, various studies have a lack of relationship between histologic type and HER2 status which is in accordance with our result [36–38]. HER2 status was not correlated to ER and PR. Our finding corroborates with that of other studies [39, 40]; however, it is different from that of several studies that showed an inverse correlation between the HER2 overexpression and the expression levels of ER and PR [37, 41–44]. The lack of relationship between HER2 status and hormonal receptor might be due to high rate ER/PR negativity resulting from preanalytical factors, including fixation deficiency. Objectively, Perou et al. [44] and Sørlie et al. [45] have revealed that HER2+ patients displayed a very low expression level of ER/PR than those of HER2- patients, indicating that patients with ER/PR+ HER2+ breast carcinoma are likely to resist hormonal therapy than those of ER/PR- HER2+ [35, 41]. Moreover, patients with ER-PR-HER2+ breast cancer have a low risk of tumor recurrence compared to ER/PR + HER2+ patients [41]. This study revealed that the HER2 overexpression was closely associated with the Nottingham grade, and thus, suggesting the aggressive pattern of the HER2+ patients with breast cancer. Several studies have confirmed this correlation [9, 37, 38, 41, 42]. In fact, HER2 oncogen is involved in various mechanisms of the normal growth of the breast epithelium. The overexpression of HER2 is responsible for an uncontrolled cell proliferation leading to breast tumors. Hereby, the HER2 positivity is associated with increased mitotic index, one of the components of Nottingham grade, which shows the aggressiveness of the breast cancer [3–7]. The overexpression of HER2, the high tumor grade, and the young age of our study population are unfavourable clinicopathologic factors which are in accordance with the literature data [9, 41, 43]. In contrast, these patients should receive anti-HER2 targeted therapy and chemotherapy which likely improve their survival.

### Limitations of the study

We have determined the HER2 status on formalin fixed paraffin-embedded breast tissue blocks regardless of tumor size, lymph node, and metastasis. As a result, we did not evaluate the correlation between HER2 positivity and these parameters.

## Conclusions

The HER2 positivity associated with relatively high grade breast carcinoma may suggest the aggressiveness of this subtype in young women. HER2-positive patients should benefit from targeted therapy that will improve their survival. The current study helps to optimise the clinical management of breast cancer patients in Ivory Coast by pointing out the well-established prognostic values of HER2.

## Abbreviations

ANOVA: Analysis of variance
ER: Estrogen receptor
HER2: Human epidermal growth factor receptor 2
IDC-NST: Invasive ductal carcinoma of no special type
IHC: Immunohistochemistry
PR: Progesterone receptor
